# The Work Continues

**DOI:** 10.1016/j.ajpc.2026.101774

**Published:** 2026-07-28

**Authors:** Michael D. Shapiro

**Affiliations:** Department of Cardiovascular Medicine, Wake Forest University School of Medicine, Medical Center Boulevard, Winston Salem, NC, 27157, USA

This is my final President’s Page.

As my term comes to an end, I have spent some time considering what I wanted to say here. The obvious approach would be to look back and provide an accounting of the past two years. There is much that could be included, and certainly much we can be proud of. But a list of accomplishments would not really capture what this experience has meant to me or what I have learned about the ASPC.

Serving as President has reinforced how fortunate I am to be part of this incredible ASPC community. Professional societies have a code of conduct, structure, and strategic plans but none of those things are what truly sustain them. They endure because people choose to give their time and energy to a shared purpose, whether it be committee participation, development of educational programs, mentorship of trainees, manuscript review, partnership building, offering of ideas, or raising concerns when they believe the Society can do better. Much of this work occurs quietly and without recognition. I saw this repeatedly throughout presidency. The people who care most deeply about the ASPC are generally the same people who are willing to do the difficult and sometimes unglamorous work required to move it forward.

I have also come to understand the presidency primarily as an act of stewardship. No president owns the Society or defines its purpose. Each of us inherits an organization that others spent many years building. We are given the privilege of guiding it for a limited period, strengthening it where we can, and then passing it to the next group of leaders. That perspective has made me appreciate even more the presidents and board members who preceded me, as well as the many people whose work will continue long after my term has ended.

Over the past two years, I have used these pages to write about several topics. A common theme emerged. Preventive cardiology is changing rapidly. The field is much broader than it was when many of us began our careers. It now routinely intersects with obesity, diabetes, kidney disease, genetics, inflammation, thrombosis, imaging, nutrition, exercise, behavioral health, and public policy. We increasingly care for patients across the lifespan, from children with inherited lipid disorders to older adults in whom treatment decisions must account for frailty, competing risks, and individual priorities.

At the same time, preventive cardiology is becoming more precise. We can identify inherited disorders earlier, characterize lipoprotein-related risk in greater detail, visualize subclinical atherosclerosis, and better recognize the different biological pathways that may lead to cardiovascular disease. Risk assessment is gradually moving beyond the use of a small number of conventional variables toward a more individualized understanding of cumulative exposure, susceptibility, and disease burden.

These developments are exciting, but they also require some caution. There is no shortage of new biomarkers, algorithms, imaging approaches, medications, devices, and digital tools. Some will meaningfully improve patient care. Others will add cost, complexity, or false precision without materially changing outcomes. The ASPC should be a place where innovation is welcomed but also examined carefully. Our field needs enthusiasm, but it also needs skepticism. We must continue asking whether a new test improves clinical decision-making, whether a treatment benefits the patients most likely to receive it, whether findings are generalizable, and whether the strength of the evidence matches the confidence of the conclusions. Preventive cardiology will be strengthened by distinguishing the advances that matter from those that merely sound promising.

The larger challenge remains translating knowledge into care. In many areas of prevention, we already know what to do. We know much about risk factors, risk assessment, lifestyle modification, and evidence-based medical therapies. Despite this understanding, implementation has remained elusive. Risk is recognized too late, effective treatment is not initiated or intensified, patients face financial, geographic, and logistical barriers, care remains fragmented, and clinicians are overwhelmed. These problems may not attract the same attention as a new biomarker or drug, but they are at least as important. Prevention that works only for patients who have access to specialists, understand how to navigate the health care system, and can afford every recommended treatment is not a successful prevention strategy.

We also need to continue moving prevention earlier. Atherosclerosis develops over decades, yet our clinical approach often waits until short-term risk is high or disease has already become evident. We need to think more carefully about lifetime exposure, inherited susceptibility, and the presence of early disease. Waiting until a person crosses an arbitrary short-term risk threshold before taking prevention seriously may mean waiting too long. Finding the appropriate balance will require better evidence, thoughtful guidelines, and clinicians who can communicate uncertainty honestly. It will also require us to move beyond rigid categories when the biology and clinical circumstances in front of us are more complicated.

One of the most encouraging parts of my presidency has been seeing how many residents, fellows, early-career clinicians, pharmacists, advanced practice professionals, and investigators are choosing to make prevention a central part of their careers. They are entering the field at an extraordinary time. They will have tools and treatments that many of us could not have imagined when we trained. They will also inherit difficult questions about access, implementation, overtreatment, undertreatment, and the proper boundaries of medical prevention. Our responsibility is to create a Society where they can question the current state of knowledge, improve upon it, and eventually replace some of our ideas with better ones. The ASPC will remain strong only if younger members see it as their professional home and believe they have a meaningful role in shaping its future.

As I conclude my term, I am grateful to the ASPC Board of Directors, our committees, our staff, our partners, and our members. I am grateful for the advice, encouragement, candor, and occasional pushback I received along the way. I am also grateful to the editors of the *American Journal of Preventive Cardiology* for providing this space. Writing these pages has periodically forced me to step away and think more carefully about the larger purpose of the work.

I am very pleased to pass the presidency to Dr. David Maron. David is a thoughtful leader, an outstanding clinician and scientist, and someone who cares deeply about prevention and about the people who work in this field. He will bring his own priorities and perspective to the role, as he should. I have great confidence in him and in the leadership team that will support him. I leave the presidency grateful for the opportunity, proud of what we accomplished together, and fully aware that much remains unfinished.

Presidents come and go, programs evolve, and scientific ideas change. However, the mission remains. We still need to identify cardiovascular risk and disease earlier, treat it more effectively, improve access to care, and ensure that scientific advances reach more than a small fraction of the people who might benefit from them. We still need to train and support the next generation. We still need to ask difficult questions, including questions about our own assumptions and practices.

My term as President is ending. My commitment to the ASPC and to this work is not.

The work continues.

